# Effects of Decapitation on Chlorophyll Metabolism, Endogenous Hormones, and Tillering Ability in *Pinus yunnanensis* Seedlings of Different Ages

**DOI:** 10.3390/biology14081070

**Published:** 2025-08-17

**Authors:** Wei Li, Xin Su, Sili Cheng, Dan Wang, Yulan Xu, Nianhui Cai

**Affiliations:** 1 Yunnan Jicheng Landscape Technology Co., Ltd., Mile 652399, China; weiliswfu8559@163.com; 2 The Key Laboratory of Forest Resources Conservation and Utilization in the Southwest Mountains of China, Ministry of Education, Southwest Forestry University, Kunming 650224, China; suxin@swfu.edu.cn (X.S.); chengsili@swfu.edu.cn (S.C.); 18208837647@163.com (D.W.); xuyulan@swfu.edu.cn (Y.X.)

**Keywords:** *Pinus yunnanensis*, decapitation, photosynthetic pigments, endogenous hormone

## Abstract

*Pinus yunnanensis* is an essential tree species in southwest China. However, its genetic degeneration problem urgently needs to be addressed. Decapitation promotes seedling propagation primarily by disrupting apical dominance, triggering hormonal changes that stimulate lateral bud growth. To investigate the response of hormones and photosynthetic pigments in *P. yunnanensis* to decapitation at different seedling ages, seedlings aged 6, 10, 14, 18, and 30 months were used as materials to carry out unified decapitation treatment, and the dynamics of photosynthetic pigments, changes in endogenous hormones, and their relationship with tillering ability were analyzed. The results showed that the photosynthetic pigments were higher in younger decapitated seedlings than in older ones, and carotenoids showed an upward trend with time. Additionally, decapitation significantly altered the balance of endogenous hormones, including the contents of GAs, ABA, SA, JA, JA-Ile, and ACC. The GA_3_ and ABA contents in the middle-aged decapitated seedlings of *P. yunnanensis* were higher. The seedlings with older decapitation ages showed higher contents of IAA, SA, and JA. Overall, seedlings with different decapitation ages exhibit significant differences in their responses to decapitation, as indicated by variations in photosynthetic pigments and hormones. This research elucidated the optimal decapitation age for *P. yunnanensis*, providing a theoretical foundation for establishing efficient decapitation nurseries and promoting near-natural propagation.

## Simple Summary

*Pinus yunnanensis* is an important timber tree species in the southwestern part of China. The problem of the degradation of its genetic resources urgently needs to be addressed. Therefore, establishing an efficient and high-quality propagation and rapid propagation system for *P*. *yunnanensis* is of great significance. In this study, decapitation treatment was carried out on *P*. *yunnanensis* at different seedling ages. The changes in hormones and photosynthetic pigments after decapitation at different seedling ages were preliminarily analyzed, and correlation analysis was conducted. It was found that six-month-old seedlings of *P*. *yunnanensis* had more balanced hormones and a higher photosynthetic pigment content, making them more suitable for topping treatment. This study provides scientific insights for selecting a appropriate topping seedling age for *P*. *yunnanensis* and for establishing efficient decapitation nurseries and promoting near-natural propagation.

## 1. Introduction

*Pinus yunnanensis* Franch. is a vital perennial afforestation tree species in southwest China. It requires ample sunlight, is drought resistant and cold tolerant, and plays a crucial role in maintaining forest biodiversity, conserving soil and water, and stabilizing ecosystems [1,2]. Currently, due to the accelerated destruction by human activities, the genetic diversity of *P. yunnanensis* is facing a severe decline, posing a threat to the local ecology [3,4].

Decapitation is a common agronomic practice that can eliminate apical dominance and accelerate the germination of lateral branches, thereby enhancing tillering capacity [5,6], creating favorable conditions for plant propagation. Several factors influence the tillering ability of *P. yunnanensis*, including the decapitation season, height, and mode [7,8,9,10]. How does decapitation specifically influence the tillering of the plant? According to previous studies, decapitation in plants can induce variations in hormones, such as indoleacetic acid (IAA), abscisic acid (ABA), cytokinin (CK), and strigolactone (SL), thereby regulating the development of tillering [11,12]. Plant hormones are fundamental regulators of plant growth, development, and architecture. Acting as central signaling molecules, they function through multi-level networks to coordinate these processes and mediate adaptive responses to environmental changes throughout plant development [13]. For instance, auxins such as IAA are involved in plant cell division, elongation, differentiation, responses to external stimuli, and apical dominance [14,15]. The influence of gibberellins (GAs) on lateral branch regulation varies among different plants, whereas reduced ABA levels promote the growth of lateral buds [16,17]. Salicylic acid (SA) and jasmonic acid (JA) coordinate with hormones, including abscisic acid (ABA) and cytokinins, to enhance plant antioxidant systems, thereby bolstering resistance to biotic and abiotic stresses [18]. Research has shown that decapitation relieves auxin-mediated suppression, enabling lateral buds to increase auxin export and subsequently initiate growth [19]. A significant decline in ABA concentrations also occurred throughout the central stem axis, with the most pronounced reduction observed in apically dominant axillary buds after decapitation [20]. To summarize, hormone balance is the core regulatory mechanism of plant propagation. Furthermore, it is worth noting that hormonal dynamics, particularly involving ABA, ethylene (ET), and JA, regulate chlorophyll degradation pathways by modulating chlorophyll-degrading enzymes, driving characteristic physiological changes [21].

The content and composition of chlorophylls, as the key photosynthetic pigment, directly determine the efficiency of photosynthesis [22]. Photosynthetic pigment dynamics govern seedling vigor, environmental adaptation, and stress responses [23]. Variations in the chlorophyll *a*/*b* ratio can reflect the optimization of photosynthetic strategies in plants as they adapt to their environment [24]. Carotenoids, as the core accessory pigments of the photosynthetic system, efficiently transfer the captured light energy and prevent photodamage to chlorophyll, also facilitating the biosynthesis of phytohormones and apocarotenoids in plants [25,26]. Previous studies have shown that decapitation of plants can delay senescence and enhance photosynthetic pigmentation and photosynthesis [27]. Furthermore, studies have also demonstrated that seedling age significantly affects chlorophyll metabolism; the photosynthetic pigments in older seedlings were considerably higher than those of younger ones [28]. Although a substantial amount of research has already been reported on plant chlorophyll, the response of hormones and photosynthetic pigments in seedlings with different decapitation ages, as well as their relationships in *P. yunnanensis,* remain unknown. Therefore, in this study, seedlings of *P. yunnanensis* aged 6 to 30 months were used as the material. The dynamic relationship between photosynthetic pigments and allometric growth with seedling age, the response of endogenous hormone content and ratio to seedling age, and its relationship with tillering were explored to provide a theoretical basis for optimizing seedling age and decapitation practices in *P. yunnanensis* cutting nurseries.

## 2. Materials and Methods

### 2.1. Plant Materials and Decapitation Treatments

*P. yunnanensis* is a major forest community species in Yunnan Province [29]. The experimental site was located in the greenhouse of Southwest Forestry University (25°04′00″ N, 102°45′41″ E), in Kunming City, Yunnan province. This region features a north subtropical semi-humid plateau monsoon climate with an average annual temperature of 14.7 ℃. The annual precipitation ranges from 700 to 1100 millimeters. The annual average relative humidity is 68 %. The soil was acidic and consisted of a low-phosphorus mixture of red soil and humus soil at a proportion of 1:2, and the soil fertility was moderate. The experimental conditions are similar to those of the ecological environment of *P. yunnanensis* seed orchards, which are suitable for the growth of *P. yunnanensis* [30]. Seedling pots measured 24 cm (top diameter) × 16 cm (base) × 20 cm (height). The seeds were collected from Midu County. The seeds were disinfected with potassium permanganate and then soaked in clean water. In January 2018, January 2019, May 2019, September 2019, and January 2020, the seeds were sown and raised. After sowing, the seedlings were nursed in the usual way during the seedling period. On 20 July 2020, seedlings of varying ages were subjected to decapitation treatment. They were 30 months old (M_30_), 18 months old (M_18_), 14 months old (M_14_), 10 months old (M_10_), and 6 months old (M_6_), for a total of 5 seedling ages. According to the previous research, the height of the stem retained after decapitation was set to 5 cm. The processed seedlings were placed evenly in the seedling bed, dividing them into 15 sections with 48 seedlings in each section, and a total of 720 seedlings were tested by decapitation (5 seedling ages, 3 replicates, 48 plants). The experimental materials are consistent with the treatment method described in reference [31].

### 2.2. Observation of Sprouting Ability

From 20 August 2020 to 20 November 2021, every 30 days after decapitation, statistics on seedling sprouting were recorded. The following were separately counted: the total number of sprouts, the number of sprouts over 1 cm, the number of sprouts below 1 cm, and the biomass of sprouts. Sprouts exceeding 1 cm were measured to the nearest 0.1 cm, while those under 1 cm were only counted.

### 2.3. Determination of Photosynthetic Pigments

The contents of chlorophyll *a*, chlorophyll *b*, and carotenoids in seedlings were measured every 60 days after decapitation, for a total of 8 periods. Each collection of sprouting needles originated from the same plant, and approximately 0.5 g of needles from three plants were collected as a sample; this was repeated three times. Photosynthetic pigments were determined by the acetone extraction method [32].

### 2.4. Determination of Endogenous Hormone Content

After 60 days of decapitation, needles in the middle part of 3 seedlings were taken as a biological repetition, and the technology was repeated three times. All the samples were collected and labeled. Suzhou Panomic Biopharmaceutical Technology Co., Ltd. (Suzhou, China, https://www.panomix.com/), determined the concentrations of hormones. The metabolite data detection system primarily consisted of ultra-high-performance liquid chromatography (UHPLC) system (Vanquish, Thermo, Waltham, MA, USA) and a high-resolution mass spectrometer (Q Exactive, Thermo, Waltham, MA, USA) (https://www.thermofisher.com/). The hormones primarily used for defense are salicylic acid (SA), jasmonic acid (JA), Jasmonic acid-isoleucine (JA-Ile), 1-aminocyclopropane-1-carboxylic acid (ACC), and abscisic acid (ABA). The auxin was indoleacetic acid (IAA) and the cytokinins included Trans-Zeatin-riboside (ZT), adenine (N6-(δ 2 N6-(Δ2-Isopentenyl) adenine, 2IP), and indole-3-pyruvic acid (IPA). Gibberellins included GA_1_, GA_3_, GA_4_, and GA_7_. Among these hormones, cytokinins, gibberellins, and auxins are all growth-promoting hormones. On this basis, the hormone content and hormone ratio were analyzed.

### 2.5. Data Analysis

The test data were statistically analyzed using SPSS 26.0, and the figures were created in Origin 2024, Tbtools-II and Adobe Illustrator 2021. Statistical analysis was conducted using one-way analysis of variance (ANOVA) and Duncan’s multiple range test. Correlation analysis was conducted using Pearson’s correlation coefficient. The allometric growth relationship between photosynthetic pigments of *P. yunnanensis* was described by the equation *y* = a*x*^b^, linearly transformed into lg*y* = lga + b·lg*x*, where *y* and *x* represent the contents of different photosynthetic pigments; a and b denote the intercept and slope between the traits, and b and 1 were compared for significant differences. If there was no significant difference between b and 1, it meant constant growth; for the contrary, it meant allometric growth [33,34,35,36,37].

## 3. Results

### 3.1. Photosynthetic Pigments in Seedlings with Different Seedling Ages

**Figure 1:**
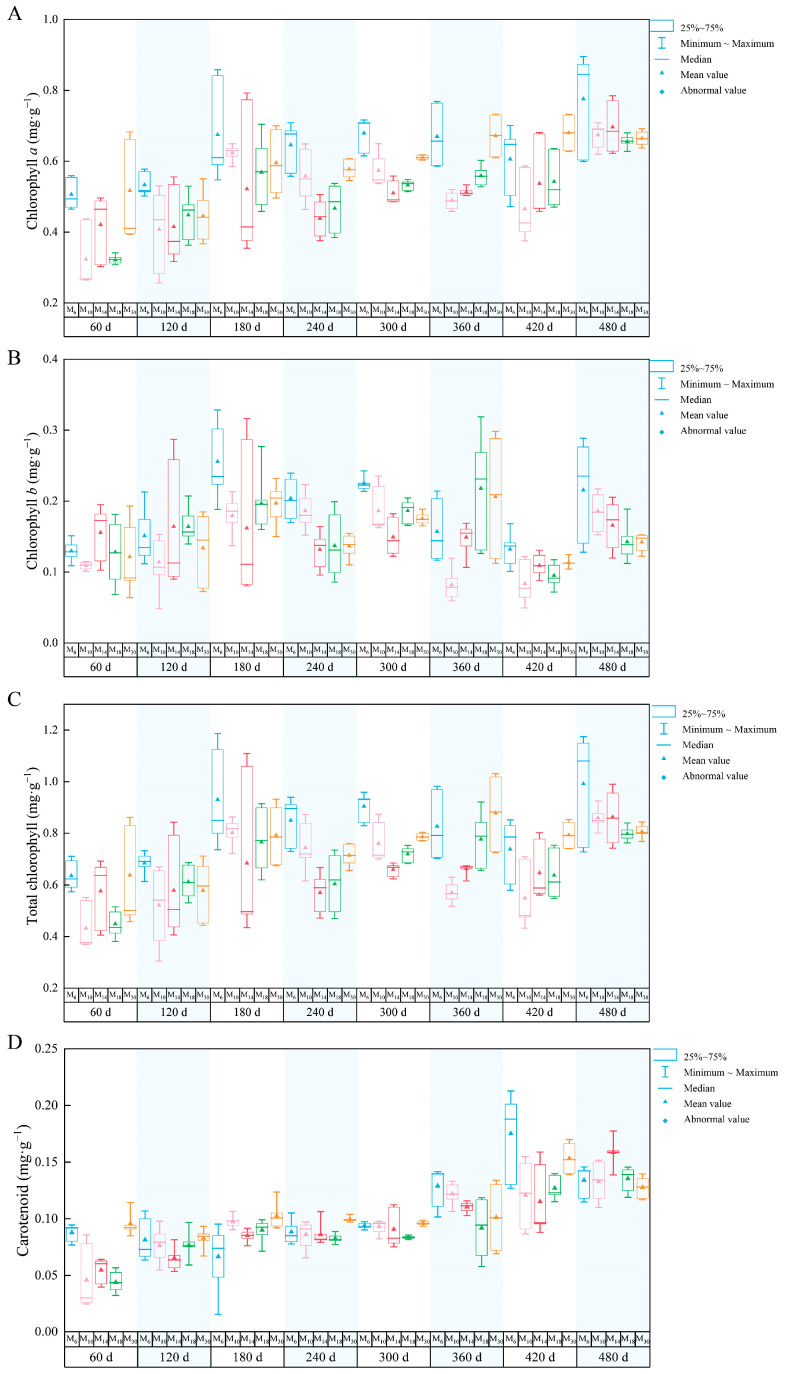
Variations in photosynthetic pigments in *P. yunnanensis* seedlings at five different ages 60–480 days after decapitation. (**A**), chlorophyll *a*, (**B**), chlorophyll *b*, (**C**), total chlorophyll (chlorophyll *a* + chlorophyll *b*), (**D**), carotenoids. The blue, pink, red, green, and orange square diagrams correspond to the five seedling ages M_6_ (6 months), M_10_ (10 months), M_14_ (14 months), M_18_ (18 months), and M_30_ (30 months) on the horizontal axis, respectively.

Decapitation of seedlings at different ages has significant effects on the total photosynthetic pigments of *P. yunnanensis*. Under different seedling ages, the trend of change in chlorophyll *a* content with time was generally consistent, and the chlorophyll *a* content of five decapitation seedlings showed an upward trend with time. (Figure 1A) The chlorophyll a content showed an upward trend after decapitation. Overall, the chlorophyll a content in M_6_ was highest at most (120 d, 180 d, 240 d, 300 d, and 480 d) sampling time points. The change in chlorophyll *b* content with time after decapitation at different seedling ages is shown in Figure 1B. The general variations in chlorophyll *b* in all the treatment seedlings showed initial increase and then decrease. Overall, chlorophyll b content was higher in seedlings that were decapitated at a younger age. As shown in Figure 1C, the total chlorophyll in plants sampled 120, 180, 240, 300, and 480 days after decapitation was highest in seedlings decapitated at 6 months of age (M_6_) compared to plants decapitated at older ages. The total chlorophyll of M_14_ was higher at 60 days after decapitation, and it remained higher at 360 and 420 days after decapitation. Overall, the total chlorophyll content in M_6_ was higher than that of other seedlings, and seedlings decapitated at five different ages showed an apparent downward trend at 240 days after decapitation, while the rest showed an upward trend as time went on. The content of carotenoids changed relatively slowly after decapitation but overall, it increased as the decapitation time prolonged.

### 3.2. Effect of the Chlorophyll a/b Ratio on P. yunnanensis Seedlings Decapitated at Different Ages

**Figure 2:**
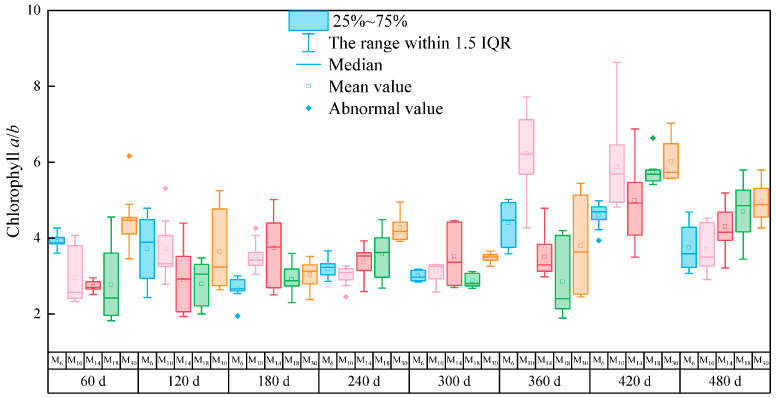
Dynamic analysis of chlorophyll *a*/*b* of *P. yunnanensis* seedlings at five different ages 60–480 days after decapitation. Note: The blue, pink, red, green, and orange square diagrams correspond to the five seedling ages M_6_ (6 months), M_10_ (10 months), M_14_ (14 months), M_18_ (18 months), and M_30_ (30 months) on the horizontal axis, respectively.

The chlorophyll *a*/*b* ratio of *P. yunnanensis* was significantly different after decapitation at different seedling ages (*p* < 0.05), as shown in Figure 2. The ratio of chlorophyll *a*/*b* in decapitated seedlings was higher at 420 days, while the ratio of chlorophyll *a*/*b* in M_10_ seedlings was the highest at 360 days after decapitation. The chlorophyll *a*/*b* ratios of M_10_, M_14_, and M_18_ were significantly lower than those of M_6_ and M_30_ at 60 days after decapitation, and there was no significant difference among seedlings with different decapitation ages at 120 days after decapitation. At 240 days after decapitation, the chlorophyll *a*/*b* ratio of M_30_ was the highest (Figure 2).

### 3.3. The Relationship Between the Relative Growth of the Photosynthetic Pigments of P. yunnanensis at Different Seedling Ages After Decapitation

**Figure 3:**
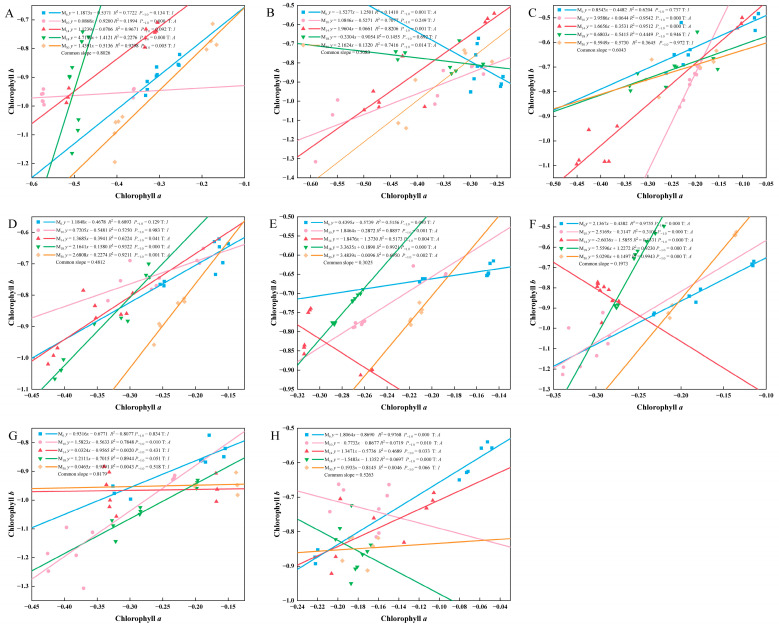
Allometric growth analysis of chlorophyll *a* and chlorophyll *b* of *P. yunnanensis* seedlings with different decapitation ages. Note: (**A**) 60 d; (**B**) 120 d; (**C**) 180 d; (**D**) 240 d; (**E**) 300 d; (**F**) 360 d; (**G**) 420 d; (**H**) 480 d. *P*-1.0 indicates the significant difference between the slope and the theoretical value 1.0, A shows the allometric growth relationship, and I denotes the isometric growth relationship. y and x represent the contents of chlorophyll *a* and chlorophyll *b*, respectively.

With the passage of time, the chlorophyll a and b of *P. yunnanensis* seedlings at different ages exhibited distinct relative growth relationships, including both allometric growth relationships and isokinetic growth relationships (Figure 3). After decapitation, the allometric growth index of chlorophyll *a* and *b* at different seedling ages showed a common slope. As time passed, the slope tended to decrease, then increase, and then decrease again. At 60 days, 180 days, 240 days, 300 days, and 420 days after decapitation, M_6_ all grew at a constant rate, and the allometric growth trajectory remained unchanged. M_18_ and M_30_ all showed an allometric growth relationship of less than 1.0 at 240 days, 300 days, and 360 days after decapitation, indicating that the growth rate of chlorophyll *a* was less than that of chlorophyll b during this period.

**Figure 4:**
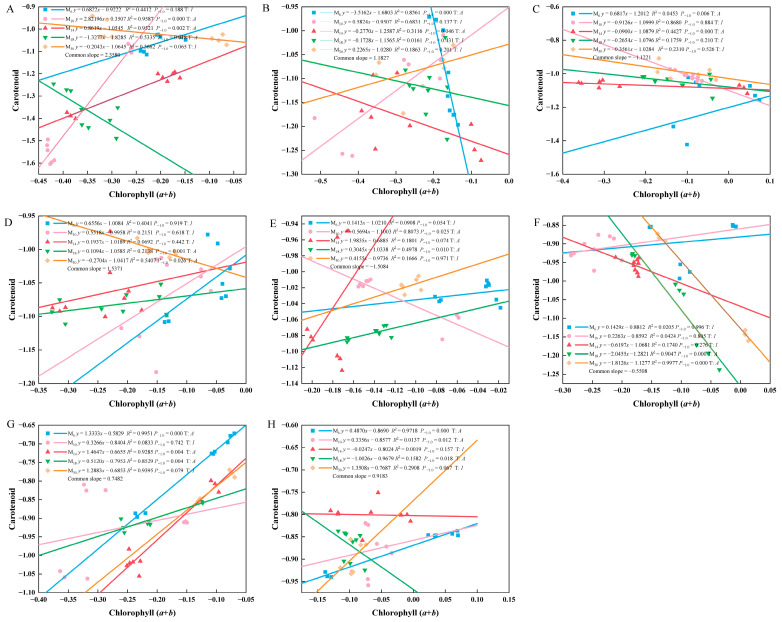
Allometric growth analysis of carotenoid and total chlorophyll (chlorophyll *a* + chlorophyll *b*) of *P. yunnanensis* seedlings with different decapitation ages. Note: (**A**) 60 d; (**B**) 120 d; (**C**) 180 d; (**D**) 240 d; (**E**) 300 d; (**F**) 360 d; (**G**) 420 d; (**H**) 480 d. *P*-1.0 indicates the significant difference between the slope and the theoretical value 1.0, A shows the allometric growth relationship, and I denotes the isometric growth relationship. y and x represent the contents of carotenoids and chlorophyll (*a* + *b*), respectively.

Over time, the relative growth relationship between carotenoid and total chlorophyll content in *P. yunnanensis* seedlings of different ages after coppicing exhibited both allometric and isometric patterns (Figure 4). The allometric index between carotenoid and total chlorophyll content across seedling ages showed a common slope during each post-decapitation period. The slope exhibited a trend of increasing, then decreasing, then increasing again, and then decreasing over time. M_10_ seedlings displayed isometric growth at 120 d, 180 d, 240 d, 360 d, and 420 d post-decapitation. M_30_ seedlings showed isometric growth at 60 d, 120 d, 180 d, 300 d, 420 d, and 480 d post-coppicing, indicating no change in the trajectory of their carotenoid-to-total-chlorophyll relative growth relationship. M_14_ seedlings exhibited isometric growth at 240 d and 360 d, while M_18_ seedlings showed isometric growth at 120 d and 180 d. At other measurement times, the relationship between carotenoid and total chlorophyll content in M_14_ and M_18_ seedlings was predominantly allometric. M_6_, M_14_, and M_30_ seedlings exhibited an allometric relationship with an index greater than 1.0 at 420 d post-coppicing. M_10_ exhibited an allometric relationship > 1.0 at 60 d, M_14_ at 300 d, and M_30_ at 480 d. This indicates that the growth rate of carotenoids exceeded that of total chlorophyll during these periods. In contrast, M_18_ seedlings never exhibited a higher carotenoid growth rate than total chlorophyll throughout the entire monitoring period after decapitation.

### 3.4. Response of Endogenous Hormone Content of P. yunnanensis at Different Seedling Ages After Decapitation

**Figure 5:**
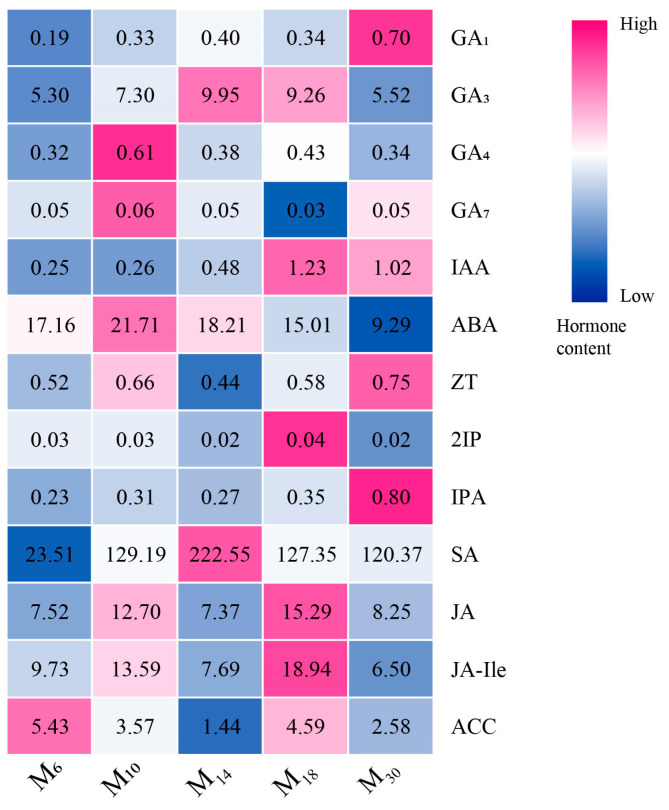
Changes in hormone content in seedlings of different ages after decapitation. Note: M_6_, M_10_, M_14_, M_18_, and M_30_ represent 6, 10, 14, 18, and 30 months of age, respectively. The value of each square represents the average specific content of hormones in the corresponding seedling age after decapitation. The closer the color of the square is to dark blue, the lower the hormone content is, while the closer the color of the square is to dark pink, the higher the hormone content is. GA_1_, GA_3_, GA_4_, and GA_7_, gibberellins; IAA, indoleacetic acid; ABA, abscisic acid; ZT, Trans-Zeatin-riboside; 2IP, N6-(δ 2 N6-(Δ2-Isopentenyl) adenine; IPA, indole-3-pyruvic acid; SA, salicylic acid; JA, jasmonic acid; JA-Ile, jasmonic acid-isoleucine; ACC, 1-aminocyclopropane carboxylic acid. The same below.

The content of endogenous hormones in *P. yunnanensis* with different decapitation ages was significantly different (*p* < 0.05), as shown in Figure 5. Except for ACC, the contents of all the hormones in M_6_ seedlings were relatively lower than in older seedlings. The contents of GA_3_ and GA_4_ in the oldest and youngest seedlings were lower than those in the seedlings of M_10_, M_14_, and M_18_, while IAA increased with decapitation age. The ABA content was the highest in M_10_ and the lowest in M_30_. The content of ZT and IPA was the highest in the M_30_ seedlings. Overall, the hormone content shows an upward trend as the age of the decapitated seedlings increases. The results showed that both growth-promoting hormones and stress hormones responded differently after decapitation, and the hormone content varied with changes in seedling age.

### 3.5. Response of the Endogenous Hormone Ratio of P. yunnanensis in Seedlings with Different Decapitation Ages

**Figure 6:**
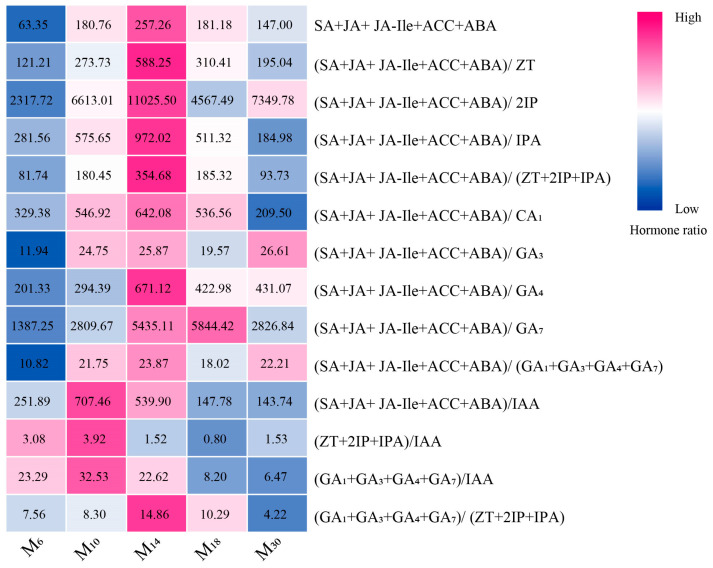
Changes in hormone ratios in seedlings of different ages after decapitation. Note: The value of each square represents the average specific hormone ratios for the corresponding seedling ages after decapitation. The closer the color of the square is to dark blue, the lower the hormone ratio is, while the closer the color of the square is to dark pink, the higher the hormone ratio is. M_6_, M_10_, M_14_, M_18_, and M_30_ represent 6, 10, 14, 18, and 30 months of age, respectively.

Hormones were classified, and their ratios were analyzed. The results showed significant differences in hormone ratios among seedlings with varying decapitation ages (*p* < 0.05). As can be seen from Figure 6, endogenous hormone ratios SA + JA + JA-Ile + ACC + ABA, (SA + JA + JA-Ile + ACC + ABA)/ZT, (SA + JA + JA-Ile + ACC + ABA)/2IP, (SA + JA + JA-Ile + ACC + ABA)/IPA, (SA + JA + JA-Ile + ACC + ABA)/(ZT + 2IP + IPA), (SA + JA + JA-Ile + ACC + ABA)/GA_1_, (SA + JA + JA-Ile + ACC + ABA)/GA_4_, (SA + JA + JA-Ile + ACC + ABA)/(GA_1_ + GA_3_ + GA_4_ + GA_7_) and (GA_1_ + GA_3_ + GA_4_ + GA_7_)/(ZT + 2IP + IPA) was the highest in M_14_, while (SA + JA + JA-Ile + ACC + ABA)/IAA, (ZT + 2IP + IPA)/IAA, and (GA_1_ + GA_3_ + GA_4_ + GA_7_)/IAA were higher in M_10_ and lower in M_30_. (SA + JA + JA-Ile + ACC + ABA)/GA_3_ was higher in M_30_. (SA + JA + JA-Ile + ACC + ABA)/GA_7_ was higher in M_18_. It can be observed that the coordination between plant hormones jointly regulates the growth and development of *P. yunnanensis* seedlings with different seedling decapitation ages.

### 3.6. Effects of Endogenous Hormone Levels on Tillering Index and Tillering Capacity of Seedlings with Different Decapitation Ages

**Figure 7:**
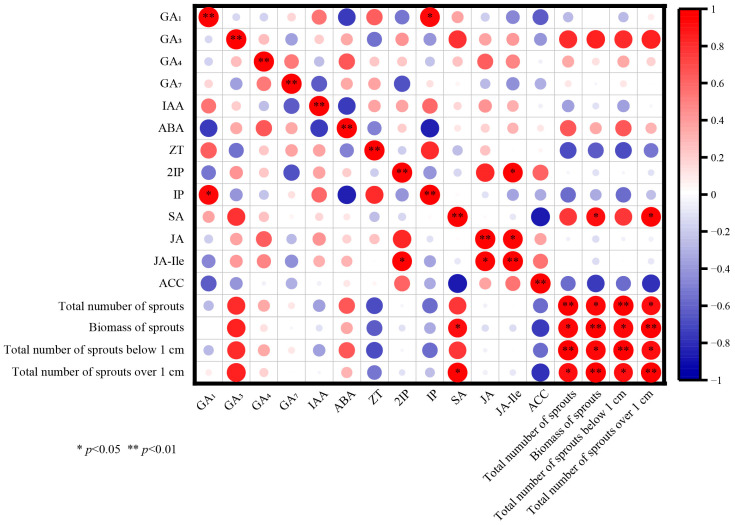
Relationships between hormone contents and sprouting index of *P. yunnanensis* seedlings.

**Figure 8:**
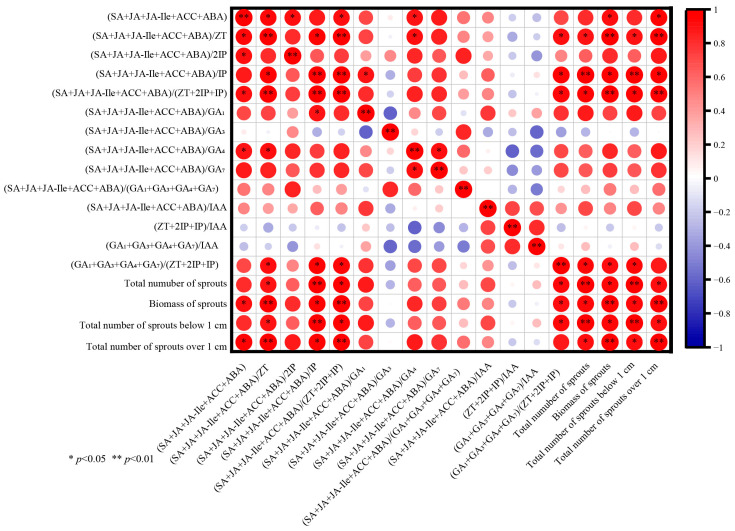
Relationships between endogenous hormone content ratios and sprouting index of *P. yunnanensis* seedlings.

As shown in Figure 7, the effects of hormone levels on the tillering index differed after decapitation. Correlation analysis revealed that GA_3_, ABA, and SA exclusively exhibited positive correlations with the biomass of sprouts and the cumulative number of sprouts exceeding 1 cm. At the same time, the remaining factors showed no significant correlation. From Figure 8, there was a specific correlation between the proportion of hormones and the number of tillers after decapitation. (SA + JA + JAIle + ACC + ABA) significantly positively correlated with sprout biomass and the cumulative number of sprouts over 1 cm, as well as (SA + JA + JA-Ile + ACC + ABA)/ZT, (SA + JA + JA-Ile + ACC + ABA)/IP, and (SA + JA + JA-ILE + ACC). Correlation analysis revealed a strong correlation between the ratio and content of endogenous hormones in *P. yunnanensis* and the sprouting index, with particularly high correlations among the ratios of endogenous hormones.

### 3.7. Principal Component Analysis of Seedling Age of Decapitation on Sprouting Index and Endogenous Hormone Ratios of P. yunnanensis

**Figure 9:**
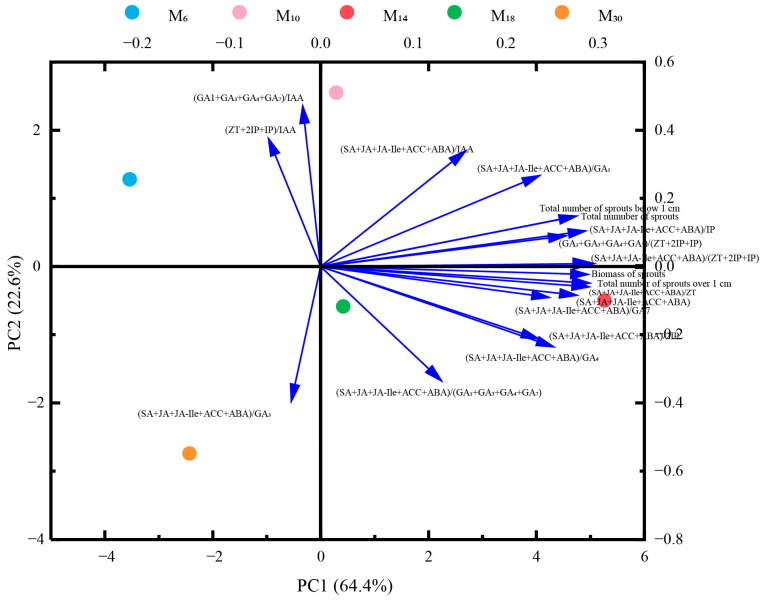
Correlations between hormone ratios and sprouting index of *P. yunnanensis* seedlings.

The 18 index data points of *P. yunnanensis* after decapitation were classified and simplified, and principal component analysis was performed. As shown in Figure 9, two principal components (eigenvalue > 1) were extracted from the 18 index data, and the cumulative variance contribution rate reached 87.0%, of which the second principal component variance contribution rate was 22.6%, which was mainly manifested in the balance between growth-promoting hormones. It mainly included (GA_1_ + GA_3_ + GA_4_ + GA_7_)/IAA, (ZT + 2IP + IPA)/IAA, (SA + JA + JA-Ile + ACC + ABA)/GA_3,_ (SA + JA + JA-Ile + ACC + ABA)/IAA, and (SA + JA + JA-Ile + ACC + ABA)/(GA_1_ + GA_3_ + GA_4_ + GA_7_). The contribution rate of the first principal component variance was 64.4%, primarily reflecting the balance between stress hormones and growth-promoting hormones. This rate also includes the other 13 indicators, excluding the second principal component analysis, which are represented by (SA + JA + JA-Ile + ACC + ABA)/IAA and (SA + JA + JA-Ile + ACC + ABA)/(GA_1_ + GA_3_ + GA_4_ + GA_7_).

### 3.8. Correlation Analysis of Seedling Age on Photosynthetic Pigments and Endogenous Hormones of P. yunnanensis Seedlings

**Figure 10:**
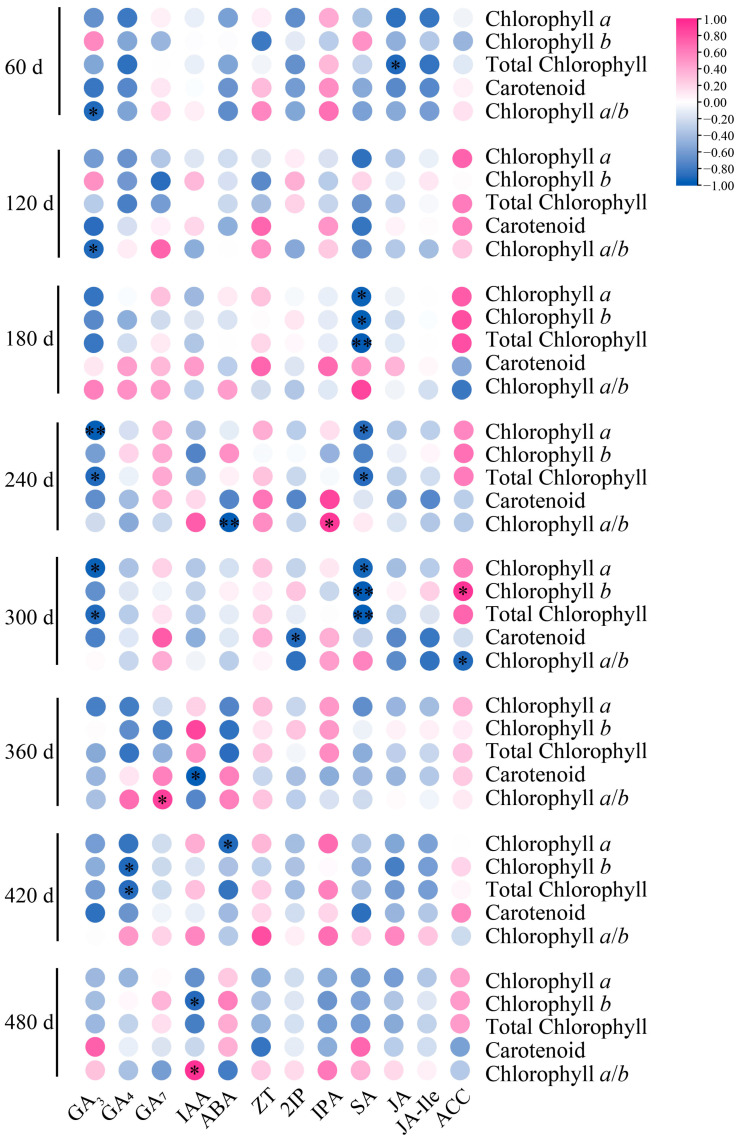
Correlation analysis between photosynthetic pigments and endogenous hormones in *P. yunnanensis* decapitated seedlings. Note: * *p* < 0.05, ** *p* < 0.01.

Correlation analysis between photosynthetic pigments and endogenous hormones in *P. yunnanensis* seedlings revealed highly significant associations between most hormone indices and photosynthetic pigment indices. Key significant correlations (*p* < 0.05) included GA_3_ negative with chlorophyll *a*/*b* (60 d, 120 d), Chl *a* (240 d, 300 d), and total chlorophyll (240 d, 300 d); GA_4_ negative with Chl *b* and total Chl (420 d); GA_7_ positive with chlorophyll *a*/*b* (300 d); GA_7_ positive with chlorophyll *a*/*b* (360 d), IAA negative with carotenoids (360 d) and Chl *b* (480 d), positive with chlorophyll *a*/*b* (480 d); ABA negative with chlorophyll *a*/*b* (240 d; *p* < 0.01) and Chl *a* (420 d); IPA positive with chlorophyll *a*/*b* (240 d); SA negative with Chl a, Chl b, and total Chl, especially at 180 d, 240 d, and 300 d; JA negative with total Chl (60 d); ACC was positively correlated with photosynthetic pigments at most measurement time points. Overall, most correlations were negative. Among the significant positive correlations, associations with the chlorophyll *a*/*b* ratio were prominent: GA_7_ (300 d), IAA (480 d), and IPA (240 d). In summary, excluding ZT and 2IP, most endogenous hormone indices exhibited significant (*p* < 0.05) or highly significant (*p* < 0.01) correlations with photosynthetic pigment indices across various developmental stages. A close, yet complex, spatiotemporal regulatory relationship exists between endogenous hormone dynamics and chlorophyll metabolism in decapitated *P. yunnanensis* seedlings, predominantly characterized by significant adverse effects, particularly during the 240- to 300-day period. Limited positive correlations primarily involved the chlorophyll *a*/*b* ratio and specific hormones (GA_7_, IAA, IPA, ACC) at distinct time points (Figure 10).

## 4. Discussion

Plant meristem development is coregulated by genetic and environmental factors, with hormones acting as central regulators [38]. Apical dominance under normal growth conditions suppresses lateral branch formation. This phenomenon occurs when auxin polar transport along the dominant main stem inhibits axillary bud outgrowth [39,40]. However, decapitation can disrupt the apical dominance through physical damage and alter the hormones within the plant, thereby regulating the development of lateral branches. For example, after decapitation, it is observed that the content of auxin in lateral buds decreases, while hormones related to defense, such as JA within the buds, increase significantly [19]. We found that the IAA content was negatively related to the cumulative number of sprouts, demonstrating that IAA was an inhibiting factor during the lateral branch germination of the plant, which was supported by a previous study [41]. And for the plants with older decapitation ages, the IAA content was higher after 60 days of decapitation. Previous research has shown that the accumulation of IAA might be related to the formation of new apical dominance [42]. Additionally, gibberellins have essential functions in regulating cell elongation, lateral branch development, and plant development [43,44]. In this study, GA_3_ exhibited a positive relationship with the number and biomass of sprouts. According to previous studies, the accumulation of GA_3_ is beneficial for *P. yunnanensis* seedlings in breaking dormancy and promoting germination [5].

Except for GAs, IAA can also cooperate with cytokinin to regulate the development of the lateral bud. The antagonism between auxin and cytokinin coordinates axillary bud development. Direct inhibition of cytokinin biosynthesis by auxin, mediated via the auxin resistance 1 (AXR1)-dependent signaling pathway, results in repressed axillary bud growth [45]. And the cytokinin produced by decapitation induction stimulates the growth of axillary buds [46]. Researchers previously revealed that the application of exogenous ZT not only helps increase the accumulation of photosynthetic pigments but also helps to regulate the homeostasis of endogenous hormones in plants under stress [47]. It was found that at most measurement time points after decapitation, the ZT content was positively correlated with the content of photosynthetic pigments. JA and its derivative JA-Ile function as signaling molecules, as well as SA, and govern plant defense and developmental responses [48,49]. Among all the treatments, the contents of JA and JA-Ile in plants at the intermediate decapitation growth stage (M_10_, M_18_), and SA in older decapitation seedlings, were higher than those in the youngest plants as well as those decapitated at older ages, indicating that selecting seedlings at an appropriate age for decapitation results in a more optimal hormonal response within the plants. Meanwhile, the content of SA was significantly positively related to the cumulative number of sprouts and the biomass of sprouts, showing that SA contributes to the abiotic defense and growth of seedlings after physical decapitation. ABA-mediated growth regulation is a very complicated process. Under appropriate concentrations, tissues, and environmental conditions, ABA has a promoting effect on plant growth and development [50]. Studies have shown that when top growth dominance is weakened, the application of moderate amounts of ABA enhances lateral bud growth [51]. A study has also shown that after decapitation, the ABA content in the axillary buds of the plant is significantly lower than that of the intact plant [52]. In this study, it was found that the plants with the oldest decapitation ages had lower ABA content. This suggests that decapitation significantly alters the ABA metabolic synthesis of plants at different growth stages. On the whole, spatial hormone distributions and finely adjusted concentration gradients orchestrate plant development [53].

Decapitation not only triggers hormonal changes but also promotes other metabolic and physiological activities in many plants, including elevated photosynthesis and chlorophyll accumulation [54]. Photosynthesis in plants is a significant physiological activity that directly affects growth, yield, and resistance to environmental adversity [55]. In the process of photosynthesis, plants mainly rely on photosynthetic pigments in leaves to absorb, transmit, and transform light energy [56,57]. Previous studies have shown that decapitation enhances plant photosynthesis [58], which may be related to improvement in the efficiency of light energy capture by the plants [59]. Following decapitation, the uppermost remaining leaves in most plants exhibit maintained or even increased chlorophyll levels [60]. In our study, chlorophyll *a*, chlorophyll *b*, and carotenoids in seedlings with different seedling ages were significantly affected. Except for M_10_, the contents of chlorophyll *a*, *b*, total chlorophyll, and carotenoids in the remaining treatments all showed an increasing trend with more days post-decapitation. And the contents of chlorophyll *a*, *b*, total chlorophyll, and carotenoids in M_6_ were higher, suggesting that seedlings with a younger decapitation age hold more potential for photosynthesis.

According to previous studies, several factors influence chlorophyll synthesis, and hormones play a crucial role in this process. For example, gibberellins, cytokinins, and JA, all of which are involved in the development and function of chloroplasts as well as in chlorophyll biosynthesis, have been shown to result in photosynthetic variations in plants [61]. Chlorophyll synthesis involves synergistic actions of multiple hormones. The various hormones interact with each other, directly or indirectly, regulating the synthesis of chlorophyll [62,63]. The variety, seedling age, and tissue location influence the accumulation of chlorophyll. It was found that the older decapitated seedlings of *P. yunnanensis* showed less accumulation of chlorophyll *a* and carotenoids overall; the same results were reported in *Manihot esculenta* Crantz [64]. Additionally, photosynthetic pigments are modulated by environmental variables including light, temperature, and abiotic stresses [23]. For instance, sufficient sunlight promotes the accumulation of chlorophyll within the plant body in summer [65]. In this study, the photosynthetic pigments of decapitated seedlings with different seedling ages showed an increasing trend 240 days to 360 days (Corresponding to March to July) after decapitation. chlorophyll *a*/*b* is of great significance in plant physiology and ecology, and can reflect the absorption and utilization of light energy by plants and the adaptability of plants to the environment [66]. It was found that for plants with younger decapitation ages, chlorophyll a / b is higher 60 to 120 days after decapitation.

## 5. Conclusions

This study investigated the responses of *P. yunnanensis* seedlings with different decapitation ages and the effects on hormones and photosynthetic pigments. The results revealed that younger *P. yunnanensis* seedlings had a more optimal hormone distribution, more photosynthetic pigments, and higher chlorophyll ratios after decapitation, indicating greater photosynthetic potential and being more conducive to the development of lateral branches. This research helps provide a reference for establishing an efficient and rapid asexual reproduction system for *P. yunnanensis*.

## Data Availability

All data generated or analyzed during this study are included in this article. All data from this study are available from the first author on request.

## References

[1] Zhu W., Liu Y., Wu J., Li C. (2025). Adaptations of *Pinus yunnanensis* Seedlings to Simulated Light Patches: Growth Dynamics and C:N:P Stoichiometry. Forests.

[2] Zhao Y., Duan X., Shu S. (2015). The Influence of Typical Forest Types on Soil Erosion Resistance in the Water Source Areas of Central Yunnan. Asian Agric. Res..

[3] Miao Y., Gao C., Li J., Liu Z., Cui K. (2023). Genetic diversity, population structure and a core collection establishment of *Pinus yunnanensis* using microsatellite markers. Eur. J. For. Res..

[4] Li Z., Gao C., Li J., Wang L., Cui K. (2024). Variation in growth traits and early evaluation of the selection of intra and interspecific hybrid progeny of *Pinus yunnanensis*. New For..

[5] Hu Z., Cheng S., He B., Tang G., Chen L., Chen S., Tang J., Xu Y., Li G., Cai N. (2025). Comparison of endogenous hormone content and balance in Pinus yunnanensis Franch. seedlings after decapitation. Front. Plant Sci..

[6] Xu T., Liu X., Wang R., Dong X., Guan X., Wang Y., Jiang Y., Shi Z., Qi M., Li T. (2016). SlARF2a plays a negative role in mediating axillary shoot formation. Sci. Rep..

[7] Zhou C., Gu X., Li J., Su X., Chen S., Tang J., Chen L., Cai N., Xu Y. (2024). Physiological Characteristics and Transcriptomic Responses of *Pinus yunnanensis* Lateral Branching to Different Shading Environments. Plants.

[8] Zhou C., Kong D., Li J., Su X., Cai N., Xu Y. (2025). The morphological and physiological responses of *Pinus yunnanensis* to different levels of shading after decapitation. Ind. Crop. Prod..

[9] Wang Y., Li J., Che F., Song Y., Wang Q., Yan T., Xu Y., Cai N., Chen S. (2023). Effects of Stumping in Different Seasons on Germination Ability of *Pinus yunnanensis*. J. Southwest For. Univ. Nat. Sci..

[10] Cai N., Hu Z., He B., Cheng S., Chen L., Tang J., Chen S., Xu Y., Li G. (2024). Response of sprouting ability of *Pinus yunnanensis* seedlings to stump-cut height. J. Northwest AF Univ. Nat. Sci. Ed..

[11] Dong L., Wu Y., Zhang J., Deng X., Wang T. (2022). Transcriptome Analysis Revealed Hormone Pathways and bZIP Genes Responsive to Decapitation in Sunflower. Genes..

[12] Fang R., Wu Y., Huang X., Hou Z., Zhang J., Wang L., Wang Y., Li Y., Chen L., Yang H. (2025). Effects of decapitation on yield-related traits of total node number per plant in soybean. Field Crops Res..

[13] Anfang M., Shani E. (2021). Transport mechanisms of plant hormones. Curr. Opin. Plant Biol..

[14] Bajguz A., Piotrowska-Niczyporuk A. (2023). Biosynthetic Pathways of Hormones in Plants. Metabolites.

[15] Li M., Wei Q., Xiao Y., Peng F. (2018). The effect of auxin and strigolactone on ATP/ADP *isopentenyltransferase* expression and the regulation of apical dominance in peach. Plant Cell Rep..

[16] Yan Y., Zhao N., Tang H., Gong B., Shi Q. (2020). Shoot branching regulation and signaling. Plant Growth Regul..

[17] Zhu J., Li X., Huang J., Wang L., Zheng Q., Li H., Chen Y., Tang J., Hao X., Wang X. (2025). Transcriptomics and plant hormone analysis reveal the mechanism of branching angle formation in tea plants (*Camellia sinensis*). Int. J. Mol. Sci..

[18] Verma K., Kumari K., Rawat M., Devi K., Joshi R. (2025). Crosstalk of Jasmonic acid and Salicylic acid with other Phytohormones Alleviates Abiotic and Biotic Stresses in Plants. J. Soil Sci. Plant Nutr..

[19] Sun D., Zhang L., Yu Q., Zhang J., Li P., Zhang Y., Xing X., Ding L., Fang W., Chen F. (2021). Integrated Signals of Jasmonates, Sugars, Cytokinins and Auxin Influence the Initial Growth of the Second Buds of Chrysanthemum after Decapitation. Biology.

[20] Everat-Bourbouloux A., Charnay D. (1982). Endogenous abscisic acid levels in stems and axillary buds of intact or decapitated broad-bean plants (*Vicia faba* L.). Physiol. Plant..

[21] Tanaka A., Ito H. (2025). Chlorophyll degradation and its physiological function. Plant Cell Physiol..

[22] Simkin A.J., Kapoor L., Doss C.G.P., Hofmann T.A., Lawson T., Ramamoorthy S. (2022). The role of photosynthesis related pigments in light harvesting, photoprotection and enhancement of photosynthetic yield in planta. Photosynth. Res..

[23] Li X., Zhang W., Niu D., Liu X. (2024). Effects of abiotic stress on chlorophyll metabolism. Plant Sci..

[24] Sonobe R., Yamashita H., Mihara H., Morita A., Ikka T. (2020). Estimation of Leaf Chlorophyll *a*, *b* and Carotenoid Contents and Their Ratios Using Hyperspectral Reflectance. Remote Sens..

[25] Zielewicz W., Wróbel B., Niedbała G. (2020). Quantification of Chlorophyll and Carotene Pigments Content in Mountain Melick (*Melica nutans* L.) in Relation to Edaphic Variables. Forests.

[26] Dhami N., Tissue D.T., Cazzonelli C.I. (2018). Leaf-age dependent response of carotenoid accumulation to elevated CO_2_ in *Arabidopsis*. Arch. Biochem. Biophys..

[27] Yordanov I., Goltsev V., Stefanov D., Chernev P., Zaharieva I., Kirova M., Gecheva V., Strasser R.J. (2008). Preservation of photosynthetic electron transport from senescence-induced inactivation in primary leaves after decapitation and defoliation of bean plants. J. Plant Physiol..

[28] Walmsley J., Adamson H. (1989). Chlorophyll accumulation and breakdown in light-grown barley transferred to darkness: Effect of seedling age. Physiol. Plant..

[29] Liu C., Liang S., Wu J., Gu J., Duan H. (2026). Response of growth and physiological-biochemical characteristics in *Pinus yunnanensis* seedlings to drought and re-watering. J. Northwest AF Univ. Nat. Sci. Ed..

[30] Luo Q., Gu L., Li S. (2025). Soil and Plant Nutrient Content in the Asexual Propagation Seed Orchard of *Pinus yunnanensis*. J. W. China Forest. Sci..

[31] Cheng S., Wang D., He B., Hu Z., Chen L., Tang J., Chen S., Xu Y., Cai N. (2024). Analysis of root morphological characteristics of *Pinus yunnanensis* seedlings at different stump-ages. J. Zhejiang AF Univ..

[32] Chao E., Wu M., Yue D., Yuan Y., Qiu N., Zhou F. (2024). Promoting effect of low concentration strontium on photosynthetic performance of Chinese cabbage seedlings: Combined leaf characteristics, photosynthetic carbon assimilation and chlorophyll fluorescence. Ecotoxicol. Environ. Saf..

[33] Tang G., Wang Y., Lu Z., Cheng S., Hu Z., Chen S., Chen L., Tang J., Xu Y., Cai N. (2024). Effects of Combined Nitrogen-Phosphorus on Biomass Accumulation, Allocation, and Allometric Growth Relationships in *Pinus yunnanensis* Seedlings after Top Pruning. Plants.

[34] Wright I.J., Reich P.B., Westoby M., Ackerly D.D., Baruch Z., Bongers F., Cavender-Bares J., Chapin T., Cornelissen J.H.C., Diemer M. (2004). The worldwide leaf economics spectrum. Nature.

[35] Cudjoe E., Bravo F., Pretzsch H., Bettinger P., Ruiz-Peinado R. (2025). Competition in mixed Scots pine and Pyrenean oak stands modifies allometry and partially affects biomass allocation during early stand development. Ecol. Indic..

[36] Li J., Yan X., Zhang P., Zhuo Z., Wang X., Huang K., Wang P., Zhou X., Ma M., Zhao Y. (2025). Multiple global change factors alter the scaling of nitrogen to phosphorus in alpine plants. Funct. Ecol..

[37] Vanneste S., Pei Y., Friml J. (2025). Mechanisms of auxin action in plant growth and development. Nat. Rev. Mol. Cell Biol..

[38] Leyser O. (2003). Regulation of shoot branching by auxin. Trends Plant Sci..

[39] Galinha C., Bilsborough G., Tsiantis M. (2009). Hormonal input in plant meristems: A balancing act. Semin. Cell Dev. Biol..

[40] Barbier F.F., Dun E.A., Beveridge C.A. (2017). Apical dominance. Curr. Biol..

[41] Shen J., Zhang Y., Ge D., Wang Z., Song W., Gu R., Che G., Cheng Z., Liu R., Zhang X. (2019). CsBRC1 inhibits axillary bud outgrowth by directly repressing the auxin efflux carrier *CsPIN3* in cucumber. Proc. Natl. Acad. Sci. USA.

[42] Chen Y., Ling Q., Li X., Ma Q., Tang S., Yuanzhi P., Liu Q.L., Jia Y., Yong X., Jiang B. (2023). Transcriptome analysis during axillary bud growth in chrysanthemum (*chrysanthemum×morifolium*). PeerJ.

[43] Wolbang C.M., Chandler P.M., Smith J.J., Ross J.J. (2004). Auxin from the Developing Inflorescence Is Required for the Biosynthesis of Active Gibberellins in Barley Stems. Plant Physiol..

[44] Okada K., Wada M., Takebayashi Y., Kojima M., Sakakibara H., Nakayasu M., Mizutani M., Nakajima M., Moriya S., Shimizu T. (2020). Columnar growth phenotype in apple results from gibberellin deficiency by ectopic expression of a dioxygenase gene. Tree Physiol..

[45] Qiu Y., Guan S.C., Wen C., Li P., Gao Z., Chen X. (2019). Auxin and cytokinin coordinate the dormancy and outgrowth of axillary bud in strawberry runner. BMC Plant Biol..

[46] Shimizu-Sato S., Tanaka M., Mori H. (2009). Auxin-cytokinin interactions in the control of shoot branching. Plant Mol. Biol..

[47] Wang D., Su P., Yang Z., Chen J., Liao R., Gao Y., Kan W., Hou J., Wu L. (2025). Combined analysis of metabolome and transcriptome in response to exogenous tZ treatment on axillary bud development in *Eucommia ulmoides* Oliver. Ind. Crop. Prod..

[48] Gomi K. (2020). Jasmonic Acid: An Essential Plant Hormone. Int. J. Mol. Sci..

[49] Spoel S.H., Dong X. (2024). Salicylic acid in plant immunity and beyond. Plant Cell.

[50] Brookbank B.P., Patel J., Gazzarrini S., Nambara E. (2021). Role of Basal ABA in Plant Growth and Development. Genes.

[51] Hartung W., Steigerwald F. (1977). Abscisic acid and apical dominance in *Phaseolus coccineus* L.. Planta.

[52] Gocal G.F., Pharis R.P., Yeung E.C., Pearce D. (1991). Changes after decapitation in concentrations of Indole-3-Acetic acid and abscisic acid in the larger axillary bud of *Phaseolus vulgaris* L. cv *Tender Green*. Plant Physiol..

[53] Binenbaum J., Weinstain R., Shani E. (2018). Gibberellin Localization and Transport in Plants. Trends Plant Sci..

[54] Van Staden J., Carmi A. (1982). The effects of decapitation on the distribution of cytokinins and growth of *Phaseolus vulgaris* plants. Physiol. Plant..

[55] Nowicka B., Ciura J., Szymańska R., Kruk J. (2018). Improving photosynthesis, plant productivity and abiotic stress tolerance—Current trends and future perspectives. J. Plant Physiol..

[56] Ye J., Liu H., Zhao Z., Xu L., Li K., Du D. (2020). Fine mapping of the QTL cqSPDA2 for chlorophyll content in *Brassica napus* L.. BMC Plant Biol..

[57] Xiong B., Qiu X., Huang S., Wang X., Zhang X., Dong T., Wang T., Li S., Sun G., Zhu J. (2019). Physiological and transcriptome analyses of photosynthesis and chlorophyll metabolism in variegated Citrus (*Shiranuhi* and *Huangguogan*) seedlings. Sci. Rep..

[58] Tschaplinski T.J., Blake T.J. (1989). Photosynthetic reinvigoration of leaves following shoot decapitation and accelerated growth of coppice shoots. Physiol. Plant..

[59] Blake T.J., Tschaplinski T.J. (1986). Role of water relations and photosynthesis in the release of buds from apical dominance and the early reinvigoration of decapitated poplars. Physiol. Plant..

[60] Satoh M., Kriedemann P.E., Loveys B.R. (1977). Changes in Photosynthetic Activity and Related Processes Following Decapitation in Mulberry Trees. Physiol. Plant..

[61] Müller M., Munné-Bosch S. (2021). Hormonal impact on photosynthesis and photoprotection in plants. Plant Physiol..

[62] Liu X., Li Y., Zhong S. (2017). Interplay between Light and Plant Hormones in the Control of *Arabidopsis* Seedling Chlorophyll Biosynthesis. Front. Plant Sci..

[63] Yan H., Fu K., Li J., Li M., Li S., Dai Z., Jin X. (2024). Photosynthesis, Chlorophyll Fluorescence, and Hormone Regulation in Tomato Exposed to Mechanical Wounding. Plants.

[64] Chaiareekitwat S., Latif S., Mahayothee B., Khuwijitjaru P., Nagle M., Amawan S., Müller J. (2022). Protein composition, chlorophyll, carotenoids, and cyanide content of cassava leaves (*Manihot esculenta* Crantz) as influenced by cultivar, plant age, and leaf position. Food Chem..

[65] Zhao Q., Wang F., Wang Y., Zhong X., Zhu S., Zhang X., Li S., Lei X., Zang Z., Tan G. (2024). Low-Temperature Regulates the Cell Structure and Chlorophyll in Addition to Cellulose Metabolism of Postharvest Red *Toona sinensis* Buds across Different Seasons. Int. J. Mol. Sci..

[66] Jinwen L., Jingping Y., Pinpin F., Junlan S., Dongsheng L., Changshui G., Wenyue C. (2009). Responses of rice leaf thickness, SPAD readings and chlorophyll a/b ratios to different nitrogen supply rates in paddy field. Field Crops Res..

